# Over-expression of FSIP1 promotes breast cancer progression and confers resistance to docetaxel via MRP1 stabilization

**DOI:** 10.1038/s41419-018-1248-8

**Published:** 2019-02-27

**Authors:** Meisi Yan, Jinsong Wang, Yanlv Ren, Lin Li, Weidan He, Ying Zhang, Tong Liu, Zhigao Li

**Affiliations:** 1 Department of Breast Surgery, Harbin Medical University Cancer Hospital, Harbin, 150000 China; 2 Department of Pathology, Harbin Medical University, Harbin, 150081 China; 3 Department of Molecular and Cellular Oncology, The University of Texas MD Anderson Cancer Center, TX 77030 Anderson, USA; 4 Department of Pathology, The University of Texas MD Anderson Cancer Center, TX 77030 Anderson, USA

## Abstract

Fibrous sheath-interacting protein 1 (FSIP1) functions centrally in breast carcinogenesis and progression, although its exact role remains to be clarified. Therefore, we sought to establish a correlation between the clinico-pathological features of breast cancer and FSIP1 expression in breast cancer tissues, as well as to validate its role in tumor progression and chemo-resistance. We analyzed FSIP1 expression in the breast cancer and para-tumor tissues by immunohistochemistry. We performed MTT, Caspase-Glo 3/7 Assay, Annexin V staining, wound healing and trans-well assays to evaluate cellular apoptosis, proliferation, migration and invasion in FSIP1 knockout and wild-type breast cancer cell lines. Additionally, we examined the effects of FSIP1 on docetaxel sensitivity in a nude mice model transplanted with control or FSIP1 knockout breast cancer cells, and also evaluate its role in tumor metastasis. FSIP1 and MRP1 interaction was determined by co-immunoprecipitation and mass spectrometry. We found that breast cancer cells and tissues consistently demonstrated elevated FSIP1 expressions, which correlated with poor overall survival. Notably, patients with high FSIP1 expression in their tumors undergoing docetaxel neoadjuvant chemotherapy had shorter disease-free survival. FSIP1 knockout in breast cancer cells significantly increased their sensitivity to docetaxel both in vitro and in vivo. Mechanistically, FSIP1 bound to the multidrug resistance protein 1 (MRP1) and stabilized it, and knocking out FSIP1 decreased MRP1 expression and increased cellular docetaxel accumulation. In sum, FSIP1 promotes breast carcinogenesis and mediates docetaxel resistance, and may serve as a novel target in the development of breast cancer therapies.

## Introduction

Breast cancer is amongst the most frequently encountered cancers globally, laying claim to being a cancer with the second highest mortality rates in women. Over 1 million diagnoses of breast cancer are made in women with 400,000 deaths due to the disease occurring annually^1^. Despite considerable progress in the treatment and diagnosis of breast cancer over the past decade, the mortality rate is still high due to frequent chemotherapeutic resistance and tumor metastasis. In depth understanding of the molecular mechanisms modulating breast carcinogenesis and drug resistance is of utmost importance in order to advance current treatment options for those with end-stage disease.

Resistance to anticancer agents is a large barrier in the successful management of multiple cancer types. Cancer cells contain ATP-binding cassette (ABC) transporter proteins, such as p-glycoprotein (P-gp), MRP1 and MRP2, that can prevent the intracellular accumulation of cytotoxic drugs via ATP-dependent efflux pumps^2^. The high expression levels of these proteins on cancer cells forms the key contributing factor in the development of chemo-resistance. Therefore, targeting ABC transporter proteins is a potential avenue to explore while innovating strategies to tackle the issue of drug resistance, and several inhibitors have been designed, such as the P-gp inhibitor, and tested in clinical trials^3^. However, results from the clinical trials have not been very satisfactory^4^, mainly due to the low binding affinity and specificity of these inhibitors^4,5^. Therefore, it is vital to identify the mechanisms and pathways of molecular regulation of the ABC transporter proteins, and find an indirect targeting strategy to overcome the conferred drug resistance.

FSIP1 is a 66 kDa intracellular protein located in chromosome 15q14. Recent experiments from our group found that FSIP1 could bind with Her2 and regulate breast cancer growth and invasiveness^6^. Other studies have reported that FSIP1 associates with PKA^7^ and SRC-3^8^, and is involved in chromosome segregation^9^. However, the exact role of FSIP1 and its underlying mechanisms in breast cancer breast cancer have yet to be reported in detail entirely.

Our study seeks to clarify the role of FSIP1 in breast cancer through analyzing the relationship between FSIP1 expression in cancer tissues and clinical features, tumor recurrence and patient survival. We retrospectively examined a breast cancer cohort in which all patients had received docetaxel-containing neoadjuvant chemotherapy. In addition, we performed mechanistic studies in in vitro and in vivo breast cancer models to further validate the role of FSIP1 in breast cancer progression and docetaxel resistance.

## Material and Methods

### Patients and breast tissue samples

A total of 404 matched pairs of breast cancer and surrounding non-cancerous tissue specimens were harvesting while patients were undergoing surgical breast cancer resection at the Harbin Medical University Cancer Hospital in Harbin, China. Consent was obtained from all subjects prior to collection and all samples were subjected to histological confirmation. The American Joint Committee on Cancer (AJCC) criteria was used to determine the clinical and tumor stage as well as clinico-pathological classification. The Elston–Ellis modification of the Scarff–Bloom–Richardson (SBR) system was used to grade tumor histologically. All experiments and protocols adhered to ethical standards outlined in the Declaration of Helsinki.

### Immunohistochemistry

Paraffin-embedded archived breast cancer tissue sections were submerged in xylene to remove the paraffin, and rehydrated with an ethanol gradient. 3% H_2_O_2_, was used to block endogenous peroxidase activity and antigen retrieval was carried out by incubating sections in a high-pressure cooker heated to1000 kW with an induction cooker. Slides were left to cool on room temperature. The sections were blocked with 5% goat serum (Beyotime Biotechnology, Beijing, China), and then incubated overnight with anti-FSIP1 antibody (Abcam) at 4 °C. After washing with phosphate-buffered saline (PBS), sections were incubated with HRP-conjugated secondary antibody (Abcam) at room temperature for 1 h. Sections were developed for visualization using diaminobenzene (DAB).

### Cell culture and lentiviral transduction

Human mammary epithelial cells (HMEC) were cultured adhering to previously published protocols^10^. Breast cancer cell lines (MCF-7, MDA-MB-435, MDA-MB-231, SKBR3 and T-47D) were cultured in a 10% fetal bovine serum (FBS) (Invitrogen, Gaithersburg, MD, USA) supplemented RPMI-1640 medium (Hyclone) with the addition of 100 mg/ml streptomycin and 100 IU/ml penicillin.

Genechem (Shanghai, China) supplied the lentivirus encoding CAS9-sgRNAs targeting FSIP1 and MRP1, and encoding MRP1 cDNA (NM_004996). Negative controls were lentivirus CAS9-sgRNA targeting green fluorescent protein (GFP).

### Animal experiments

Animals were maintained and handled with the permission of the Administrative Panel on Laboratory Animal Care of the Harbin Medical University Cancer Hospital. Breast cancer cells transduced with the lentiviruses were subcutaneously injected (5 × 10^6^ cells in 100 ml PBS) into the flanks of BALB/C nude mice. Weekly images of mice were obtained with the Kodak In-Vivo FX Pro (Kodak, New York, USA), using the fluorescent intensity to measure GFP + xenograft tumor volume. Shortly before taking these images, mice were anesthetized with phenobarbital sodium. Imaging parameters are as follows: an emission wavelength of 535 nm. An excitation wavelength of 490 nm and exposure times of 1 to 2 min.

For inducing lung metastasis, 5 × 10^6^ breast cancer cells in a 100 ml PBS suspension were injected into the tail veins and metastases were monitored every 7–10 days by measuring the bioluminescent signals. 5 min before capturing these images, 150 mg/kg of intraperitoneal D-luciferin solution was administered to the mice. Images were obtained at exposures of 10 to 30 s in duration. After 7 weeks, mice were sacrificed for further evaluation.

For docetaxel (Sigma) response experiments, 5 × 10^6^ wild type or FSIP1 knockout MDA-MB-231 cells were inoculated into nude mice. Ten days after implantation, the mice were given either vehicle or 25 mg/kg docetaxel once weekly. For doxorubicin experiment, the treatment was given from day 5 after tumor inoculation at the dose of 8 mg/kg weekly. Tumor volumes were monitored every 5 days and the tumor regression rate was calculated as: (sgNC tumor volume = sgNC docetaxel tumor volume) / sgNC tumor volume × 100%.

### Histological analyses of xenograft tumors

Mice were humanely sacrificed after the last session of in vivo optical imaging. Lung tissues were dissected, fixed, and paraffin-embedded for further histopathological analysis. Tumor tissues were stained with hematoxlyn and eosin (H&E) staining prior to imaging.

### RNA extraction and quantitative real-time PCR

The TRIzol® Reagent (Invitrogen, CA, USA) was used for extraction of cultured cell lines’ total RNA which was then reverse transcribed into cDNA with a specific stem-loop real-time PCR miRNA kit (RiboBio, Guangzhou, China). The SYBR Green qPCR system (Takara, Dalian, CHN) was utilized in the a/1uantitative real-time PCR (qPCR) procedure with GAPDH acting as an internal control. qPCR was carried out with the Applied Biosystems 7900HT real-time PCR system.

### Western blotting

Cells were lysed in a buffer comprised of 1 mM Na2-VO4, 2 mM EDTA, 50 mM Tris (pH 8.0), 120 mM NaCl, 0.5% Triton X-100, and 1:300 protease inhibitor cocktail (P8340; Sigma-Aldrich). Extracted proteins were separated by sodium dodecyl sulfate (SDS)–polyacrylamide gel electrophoresis (PAGE) and applied onto a primary antibody PVDF membrane. The membranes were incubated with primary antibodies (Sigma, St Louis, MO, USA) and subsequently horseradish peroxidase (HRP)-conjugated secondary antibody (Abcam). Band detection was performed with the Supersignal West Pico ECL chemiluminescence kit (Pierce) and images printed ont0 Kodak X-ray film (Eastman Kodak Co, NY, USA). Antibody information: anti-FSIP1 (H00161835-M02, Abnova); anti-MRP1 (SC-18835, Santa Cruz); anti-GAPDH (SC-32233, Santa Cruz); anti-MDR1 (#12683, Cell Signaling); anti-BAX (#2772, Cell Signaling); anti-Cleaved Caspase-3 (#9664, Cell Signaling); Cleaved PARP (#5625, Cell Signaling).

### MTT assay

Lentiviruses were left to infect cells overnight before cells were transferred onto a six-well plate at the density of 10^5^ cells/well. The 3-(4, 5-methylthiazol-2-yl)-2,5-diphenyltetrazolium bromide (MTT) colorimetric assay was used to evaluate cell growth for 5 days. Each day, 50 μg MTT solution was added per well and the cells were incubated for 4 h. Acidic isopropanol was used to solubilize intracellular MTT with the the optical density was measured at 570 nm. Time taken for number of cells to double was determined during the phase of exponential growth. Three independent experiments were performed, and each condition was assayed in triplicates.

### Caspase-Glo 3/7 assay

The assay was performed following the manufacture. Briefly, cancer cells was treated with docetaxel at indicated concentration for 72 h. CellToxTM Green was added at time of treatment. At 72 h Caspase-Glo 3/7 reagent was added and plates were shaken for 30 min. Fluorescence signal was read at 485 nm excitation, 520 nm emission, followed by luminescence.

### Annexin V-FITC apoptosis assay

The Annexin V-FITC (ebioscience, 88–8007) and PI cell staining were performed as per the manufacturer’s instructions to determine cell apoptosis, and flow cytometry (FACSCalibur, Becton Dickinson) was used to determine percentage of apoptotic cells.

### Immunofluorescence

Cells cultured on cover slips were fixed with 4% paraformaldehyde, permeabilized with 0.25% Triton and left exposed to primary antibodies overnight at 4 °C. The next day, cells were re-incubated with followed rhodamine-conjugated goat anti-rabbit IgG (abcam, Cambridge, UK). DAPI was used to counterstain the nuclei, and cells were then visualized with the confocal laser scanning microscope (Olympus FV1000, Olympus, and Center Valley, USA).

### Cell motility assay

To determine the ability of cells to migrate, 8 × 10^5^ cells were seeded onto 60-mm dishes and left for 24 h to grow. A linear wound was inflicted by scratching the confluent cell monolayer with a pipette tip. The distance between two wound edges was evaluated by three random wound field photographs both immediately after the wound and 24 h after the procedure and the degree of wound closure was taken as an indicator of cell motility^11^.

### Cell migration and invasion assays

Cell lines were assessed for their invasive and migratory capabilities on an 8-mm pore size 24-well Transwell plate (Corning, New York, USA). For the migration assay, 2.5 × 10^4^ cells were placed on the uppermost chamber that contained a non-coated membrane. For the invasion assay, the chamber slots were prepared by first applying a coat of 200 mg/ml Matrigel, and left overnight to dry. The top chamber then received 5 × 10^4^ cells. Cells in both assays were suspended in serum free medium, with the lower chamber of both Transwell plates containing serum supplemented medium which functioned as a chemo-attractant. After a 24-h period of incubation at 37 °C, cells on the top chambers were considered to be non-invasive or non-migratory and were disposed of by gentle cleaning with cotton. Cells present on the bottom chambers were considered to be invasive, and were removed for fixation in 100% methanol for 10 min, air-dried, stained in 0.1% crystal violet, and quantified with a microscope (Ti-E, Nikon, Tokyo, JP). At least three independent assays were performed for each experimental condition.

### Co-immunoprecipitation (Co-IP) of FSIP1 and MRP1 in breast cancer cells and mass spectrometry

MDA-MB-431 cells that were stably infected with FSIP1 (His-tagged) lentiviral vector or empty vector was subjected to radio-immunoprecipitation assay (RIPA) buffer supplemented with proteinase inhibitor (Novagen, Darmstadt, Germany) in order to extract its proteins. The resultant cell lysate (containing 100 μg protein) was incubated with anti-His-tag (Abcam, Cambridge, Massachusetts, USA) or IgG (as a negative control) (Santa Cruz, Dallas, Texas, USA). Whole cell lysate (150 μg protein) and the immuno-precipitant of anti-His-tag, anti-MRP1 antibody or IgG were immunoblotted with either anti-FSIP1 or anti-MRP1 antibody to confirm the interaction of FSIP1 and MRP1. The lysate (6% input, 10 μg protein) was also used as a control. To validate the Co-IP results, immune complexes were then centrifuged to encourage precipitation before undergoing SDS-PAGE gel separation. Gel wells positive for candidate protein targets were extracted, subjected to in-gel digestion and subsequently underwent matrix-assisted laser desorption/ionization time-of-flight/ time-of-flight (MALDI-TOF/TOF) mass spectrometry^8^.

### High-performance liquid chromatography (HPLC)

A total of 6 × 10^4^ lentivirus-infected cells were seeded into each well of a six-well culture plate and underwent a two-hour incubation with 1 mg/l docetaxel. HPLC (Supelco Co Ltd, PA) analysis was performed on a 1200 system using a diamond C18 reversed-phase column (4.6 mm × 250 mm). Methanol and water (55:45, v/v) with potassium dihydrogen phosphate (0.06 M) were used in the mobile phase, with a flow rate of 0.8 ml/min and pH adjusted to 5.0. 20 ml samples were injected and detection performed at 297 nm wavelength.

### Statistical analysis

The JME Pro10 software and Graphpad Prism 7 were used to carry out statistical analyses. Data were presented as an average of independent experiments ± SD or SEM. Various statistical tests such as Fisher’s exact test, Pearson’s *Χ*^2^ test, Student’s t test, one-way ANOVA and two-way ANOVA were utilized based on the type of data present. Survival curves and disease-free survival curves were plotted with the Kaplan–Meier method, and univariate analysis was performed with log-rank tests. The Cox multivariate proportional hazard regression model was used to conduct multivariate analysis. Statistical significance was attained when *p* < 0.05. *p < 0.05, **p < 0.01, ***p < 0.001.

## Results

### FSIP1 is over-expressed in breast cancer cell lines and tumor tissues

**Fig. 1:**
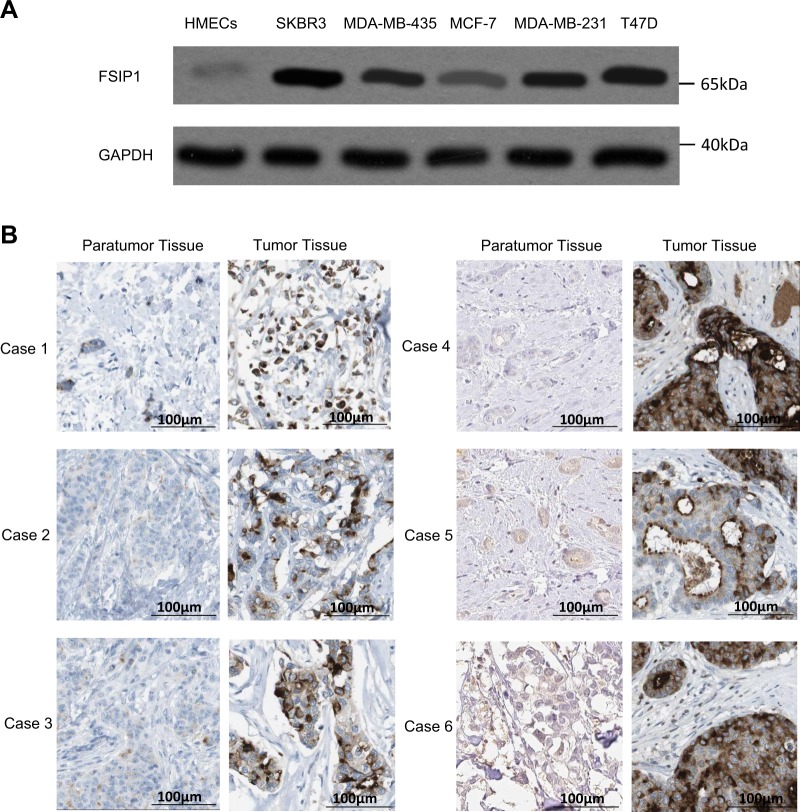
FSIP1 is over-expressed in breast cancer cell lines and tumor tissues. **a** FSIP1 expression in breast cancer cell lines and normal cell line detected by western blotting. **b** FSIP1 expression in 6 pairs of breast cancer and adjacent non-cancerous tissues as visualized by IHC

FSIP1 protein expression markedly increased in every breast cancer cell line when contrasted to the HMECs (Fig. 1a). Furthermore, comparative analysis of 6 pairs of tumor and para-tumor tissues revealed that FSIP1 expression levels were notably higher in breast cancer tissues than in the matched surrounding healthy tissues (Fig. 1b). These findings indicate that breast cancer tissues display upregulated FSIP1 expression, alluding to this gene’s potential oncogenic role.

### FSIP1 overexpression is associated with shorter overall survival and poor therapeutic efficacy of docetaxel

**Fig. 2:**
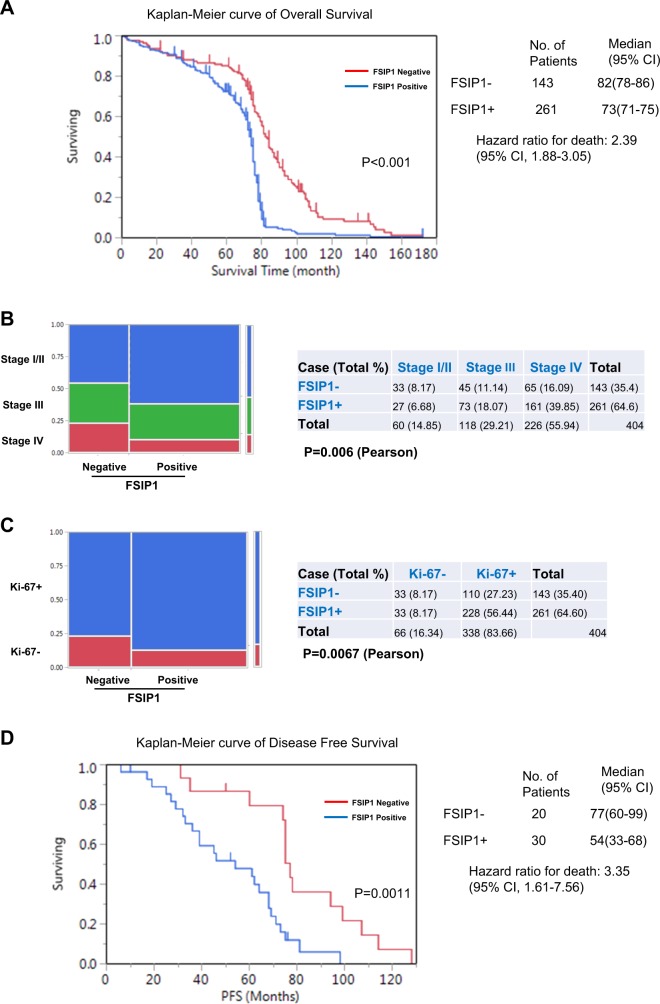
High FSIP1 expression is correlated with high malignancy, shorter overall survival and poor therapeutic response to docetaxel in breast cancer patients. **a** Kaplan–Meier curves depicting the overall survival of two patient cohorts stratified by FSIP1 expression. **b**, **c** FSIP1 overexpression correlates with higher disease stage and high Ki67 levels. **d** Disease-free survival in patients under docetaxel-based neoadjuvant therapy

To investigate the correlation between FSIP1 and the clinico-pathological features of breast cancer, FSIP1 expression was examined in 404 breast cancer samples stored in paraffin blocks. These tissue samples comprised of from 60 cases of clinical stage I / II (14.85%), 118 cases of stage III (29.21%), and 226 cases of stage IV (55.94%) breast cancer. Statistical analysis uncovered a correlation between high FSIP1 levels and more advanced disease (Fig. 2b. *p* = 0.006. Pearson) as well as high Ki-67 expression levels, a marker of cellular proliferation. (Fig. 2c, *p* = 0.0067. Pearson). Consistent with previous studies^6^, FSIP1 expression appeared to correlate positively with Her2 expression (*p* = 0.043. Pearson, data not shown). Nevertheless, statistical analysis did not support any associations between FSIP1 expression and other parameters like age, P53 status, ER status, PR status and menopause status.

The Kaplan–Meier method and the log-rank test were utilized to investigate the relationship between FSIP1 expression and overall patient survival rates. Patients who had higher levels of FSIP1 were also found to have overall survival times that were significantly shorter (median overall survival time, 73 vs. 82 months, *p* < 0.001; Fig. 2a). Multivariate Cox proportional hazard regression revealed high FSIP1 expression to be an independent prognostic marker for overall survival (*p* < 0.001; HR = 2.39; 95% CI 1.88–3.05).

In addition, we have checked breast cancer database in TCGA as an independent cohort. In Nature. 2012 Database^12^, there are totally 825 samples in which 2.6% patients (*n* = 27) had FSIP1 mRNA downregulation (Supplementary Figure. S1). The overall survival demonstrated that low FSIP1 mRNA patients showed improved overall survival (Supplementary Figure. S1). Although the p value is 0.19 and it is not statistics significant, the trend is very obvious. Since the number of patients whose FSIP1 mRNA level were defined as low expression is very small (2.6%), it is reasonable that there is no significant *p* value.

The relationship between FSIP1 expression and disease-free survival (DFS) was analyzed in one cohort where the patients had received docetaxel-based neoadjuvant. High levels of FSIP1 correlated with shorter DFS time (median DFS time, 54 vs 77 months, *p* = 0.0011; Fig. 2d), and similarly, high FSIP1 was also found to be an independent prognostic indicator of DFS, as revealed by multivariate Cox proportional hazard regression analysis (*p* = 0.0016; HR = 3.35; 95% CI: 1.61–7.56). These results indicated that FSIP1 may play an oncogenic role and promote docetaxel resistance in breast cancer.

### FSIP1 knockout suppresses breast cancer cell invasion and viability

**Fig. 3:**
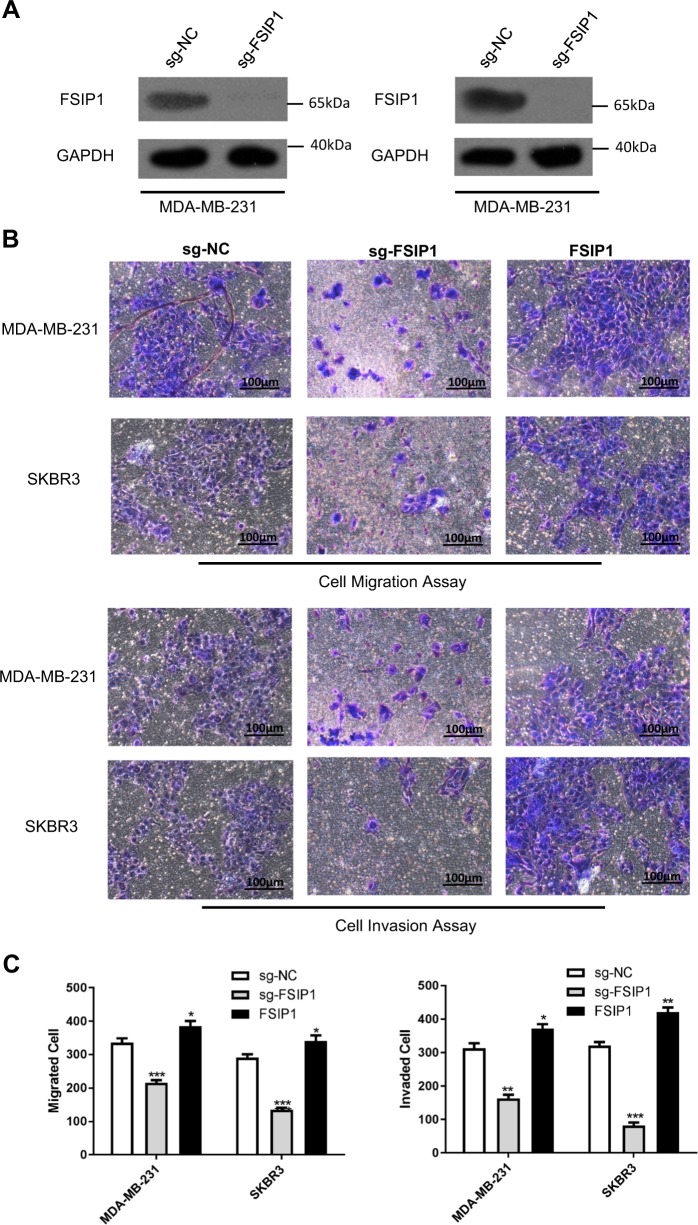
FSIP1 knockout cancer cells show attenuated invasion abilities. **a** Western blotting confirmed FSIP1 knockout in the relevant cell lines. **b** Representative images of the cell migration assay and invasion assays **c** Quantification of cell invasion and migration

**Fig. 4:**
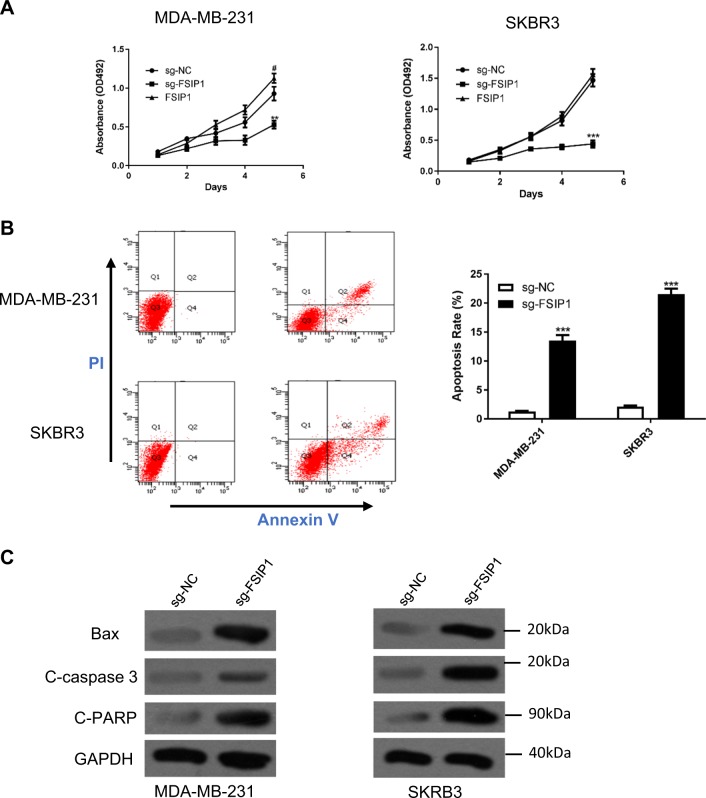
FSIP1 knockout decreases breast cancer cell viability and promotes apoptosis. **a** MTT assay showing the proliferation of the wild type, FSIP1 knockout and FSIP1 re-expressed SKBR3 and MDA-MB-231 cells. **b** Apoptosis was measured by the flow cytometry. **c** Apoptosis markers were detected by western blotting

**Fig. 5:**
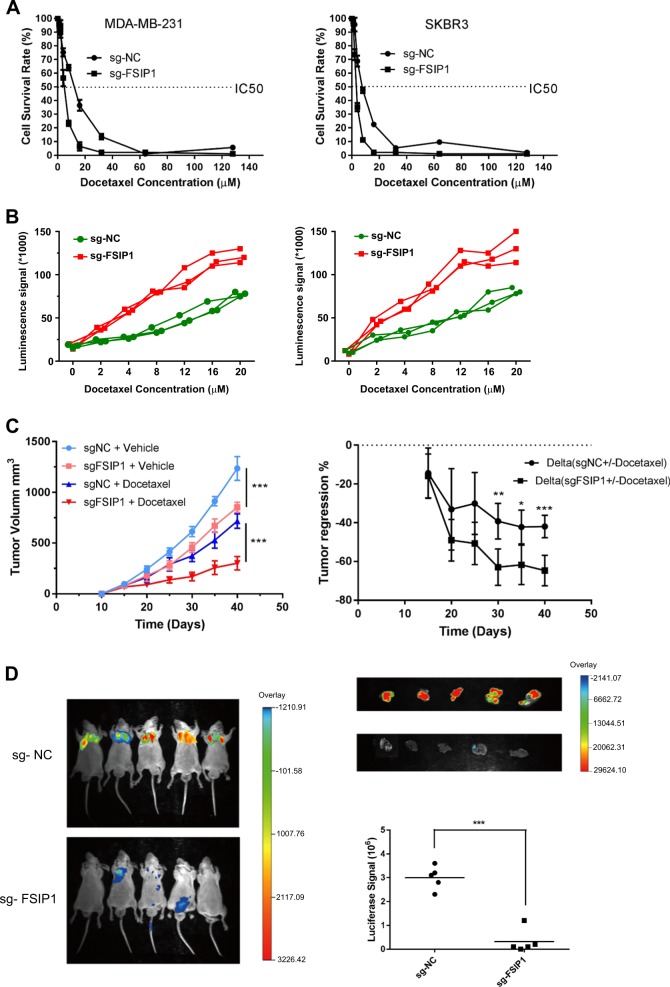
FSIP1 knockout sensitizes breast cancer cells to docetaxel. **a** MTT assay showing the IC50 of docetaxel in control and FSIP1 knockout MDA-MB-231 and SKBR3 cells. **b** Caspase-Glo 3/7 Assay to test the docetaxel sensitivity in MDA-MB-231 wild-type or FSIP1 knockout cells. **c** Tumor volumes in nude mice transplanted with control and FSIP1 knockout MDA-MB-231 cells and regression in tumor volumes post docetaxel administration. The *** indicates a statistical significant difference in tumor volume exists between sgNC + Docetaxel group and sgFSIP1 + Docetaxel group. **d** Bioluminescence imaging showing lung metastasis in the mice receiving either sgNC- or sgFSIP1-Luc-MDA-MB-231 via tail vein injection

To further elucidate the oncogenic function of FSIP1, the CRISPR/CAS9 recombination system was used to knockout FSIP1 in the high expression cell lines MDA-MB-231 and SKBR3. The knockout of FSIP1 protein in these cells was validated by Western blotting (Fig. 3a), and significantly inhibited cell invasion and migration (Fig. 3b, c). Exogenous expression of FSIP1 rescued the cells from the effects of knockout. Furthermore, FSIP1 knockout significantly reduced MDA-MB-231 and SKBR3 cell proliferation while increasing apoptosis (Fig. 4a, b). Increased levels of the apoptotic proteins Bax, cleaved-caspase 3 and cleaved-PARP in FSIP1 knockout cells also supported the anti-apoptotic function of FSIP1. FSIP1 knockout MDA-MB-231 derived tumors showed regressed growth compared with those derived from control cells in the nude mice (Fig. 5b. light blue VS light red). Interpreted as a whole, these findings strongly allude to the central role that FSIP1 may have in breast cancer carcinogenesis.

### FSIP1 knockout sensitizes breast cancer cells to docetaxel

We proceeded to evaluate changes in breast cancer cell sensitivity to docetaxel in the presence of altered FSIP1 expression. FSIP1 knockout significantly lowered the IC50 and increased the sensitivity of SKBR3 and MDA-MB-231 cells to docetaxel (Fig. 5a).

To examine the in vivo effect of FSIP1 on breast tumor growth, nude mice were subcutaneously injected with FSIP1-sgRNA or GFP-sgRNA transduced MDA-MB-231 cells. Transfected mice were administered either docetaxel or vehicle control, with subcutaneous tumor growth monitoring performed for each group over the next 7 days. The difference between the tumor volumes in the vehicle treated and docetaxel treated mice (Δ value) were calculated for both FSIP1-sgRNA and GFP-sgRNA derived xenograft cohorts. As shown in Fig. 5b, the tumor regression effect of docetaxel was significantly higher in mice injected with FSIP1 knockout breast cancer cell, indicating that FSIP1 mediates docetaxel resistance.

FSIP1-mediated docetaxel resistance was also tested in animal models of lung metastasis. Nude mice that were under docetaxel treatment were injected with either FSIP1-knockout or control luc-MDA-MB-231 cells through their tail veins. The mice were monitored weekly by an optical bioluminescence imaging machine. After 7 weeks of monitoring, the mice were sacrificed and their lungs were dissected and evaluated for bioluminescence signals. Significant lung metastasis was seen in all mice (5 of 5) who received vector cells, while tumor cells had not metastasized to the lung in any of the mice transplanted with the FSIP1 knockout cells (Fig. 5c).

### FSIP1 binds to and stabilizes MRP1 and confers docetaxel resistance via MRP1

**Fig. 6:**
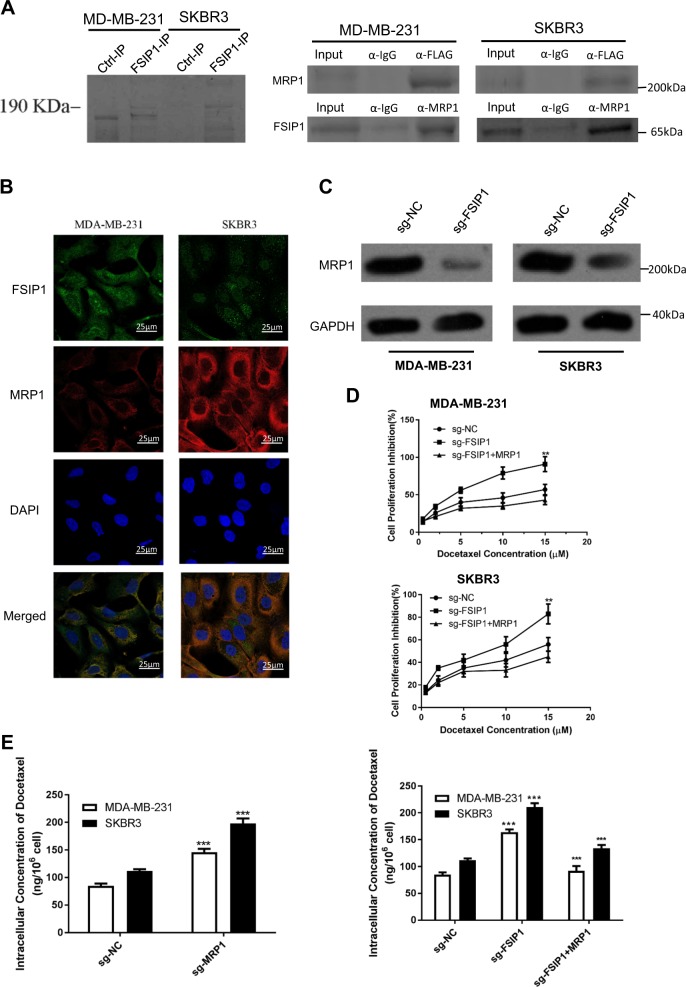
MRP1 can bind and be stabilized by FSIP1, and is responsible for FSIP1-mediated docetaxel resistance. **a** Co-IP and MS analysis identified the binding of FSIP1 and MRP1. **b** Confocal images showing the membrane-proximal co-localization of FSIP1 and MRP1. **c** FSIP1 knockout can decrease MRP1 levels. **d** MTT assay showing that overexpression of MRP1 in FSIP1 knockout cells can reverse the effect of knockout. **e** HPLC Analysis showing increased intracellular accumulation of docetaxel in FSIP1 knockout cancer cells, which was attenuated by MRP1

To gain insights into the molecular mechanisms of FSIP1-mediated docetaxel resistance, we first identified molecules that may potentially interact with FSIP1 with Co-IP and protein mass spectrometry. Comparison of the anti-His immuno-precipitates of MDA-MB-231 cells over-expressing FSIP1-His with MDA-MB-231 cells infected with control vector (pcDNA3.1/His), or with the anti-IgG immuno-precipitates indicated that MRP1 interacts with FSIP1 (Fig. 6a, left panel).

We then performed Co-IP with FSIP1 antibody in MDA-MB-231 and SKBR3 cells in order to confirm the physical interactions between FSIP1 and MRP1. In both cell lines, FSIP1 co-precipitated with the MRP1 antibody (Fig. 6a, right panel). Confocal microscopy images showed that FSIP1 was mainly localized to the membranes and nuclei of SKBR3 and MDA-MB-231 cells, and MRP1 was found in both the cytoplasm and nucleus and was co-localized with FSIP1 in the membrane (Fig. 6b).

The role of MRP1 in FSIP1-mediated oncogenic function was also analyzed. FSIP1 knockout significantly reduced the levels of MRP1 in breast cancer cells, and MRP1 overexpression in the FSIP1 knockout SKBR3 and MDA-MB-231 cells significantly attenuated docetaxel sensitization and lowered intracellular docetaxel accumulation (Fig. 6d). We also tested whether FSIP1 regulates MRP1 is specific, we tested whether FSIP1 knocking out can affect another drug-resistance-related protein MDR1 expression. We found that FSIP1 knocking out does not affect MDR1 expression (Supplementary Fig S2), indicating FSIP1 specifically regulates MRP1 and mediates drug resistance.

Since the substrate of MRP1 is broad, we tested how FSIP1 affects the sensitivity of another active agent in breast cancer therapy - doxorubicin. We found that FSIP1 knockout can sensitive breast tumors to doxorubicin treatment (Supplementary Fig. S3). Taken together, FSIP1 mediates docetaxel resistance in breast cancer, at least in part, via MRP1.

## Discussion

Principal discoveries in these series of experiments are FSIP1 overexpression is significantly associated with increased malignancy, invasiveness, and docetaxel resistance of human breast cancer cells, and that knocking out FSIP1 decreased the proliferation and migration, and increased the susceptibility of breast cancer cells to chemotherapeutic agents. Furthermore, MRP1 was identified as a potential functional partner that physically interacted with FSIP1, and responsible for FSIP1-mediated docetaxel resistance.

Carcinogenesis or malignant transformation of cells is a multi-step process requiring reprogramming of energy metabolism, insensitivity to growth-inhibitory signals, unrestrained growth signals, limitless replicative potential, apoptosis, evasion of immune detection, tissue invasion and metastasis and sustained angiogenesis^13,14^. To characterize the function of FSIP1 in breast cancer where it is frequently upregulated, CRISPR/CAS9 based lentivirus was used to knockout FSIP1 in two breast cancer cell lines SKBR3 and MDA-MB-231 cells, which have a high endogenous expression of FSIP1. Knocking out FSIP1 significantly attenuated cell growth, invasion and migration, as well as augmenting breast cancer cell sensitivity to docetaxel. Additionally, both subcutaneous xenograft and lung metastatic models validated the hypothesis that FSIP1 inhibition significantly repressed tumor growth whilst augmenting docetaxel sensitivity in the nude mice. Consistent with this, overexpression of FSIP1 was significantly correlated with poor docetaxel response in breast cancer tissues.

Drug resistance in cancers is thought to be mediated by the multidrug resistance associated protein 1 (MRP1), which functions as a cellular transporter of physiological substrates as well as a broad range of therapeutic agents^15^. Many chemotherapeutic agents, including docetaxel, have been identified as MRP1 substrates^16–18^. We performed Co-IP of FSIP1 followed by protein sequencing and identified MRP1 as the FSIP1 interacting partner. Although multidrug transporter proteins induce chemo-resistance by enhancing the efflux of anticancer drugs from cancer cells, the therapeutic targeting of ABC transporters has been unsuccessful^4,5^ due to low binding affinity or low specificity of the inhibitors. An alternative strategy is to target the upstream components that regulate the stabilization of these transporter proteins. We have shown that the oncogenic FSIP1 can also interact with and stabilize MRP1, and likely mediates docetaxel resistance via this mechanism. Therefore, targeting FSIP1 can be a “one stone two birds” strategy since blocking FSIP1 inhibits cancer cell proliferation and promotes apoptosis^6^, and can also destabilize MRP1 and sensitize cancer cells to chemotherapeutic agents. However, we did not elucidate the underlying mechanisms of FSIP1-mediated stabilization of MRP1, which will be addressed in future studies.

Additionally, Although MRP1 mediated drug-resistance has been recognized in cancer therapy^19^, there is some clinical data that does not support that high expression of MRP1 can be a biomarker to indicate resistance to chemotherapy in breast cancer. For example, Moureau et al. studied impact of MRP1 mRNA levels on 5-year disease-free survival (DFS) and overall survival (OS) of 171 patients by univariate and multivariate Cox analysis^20^. They stated that they did not found a correlation. However, Filipits et al. detected MRP1 expression by IHC and found that high MRP1 expression promotes the clinical resistance to adjuvant chemotherapy in 516 patients^21^. We believe that the different methods (RT-PCR v.s. IHC) of MRP1 detection may be one reason to make it controversial. Therefore, a large prospective cohort study with a consistent MRP1 detection method may be needed to justify MRP1 role in chemotherapy resistance.

## Conclusions

We identified a potential oncogene FSIP1, whose expression is commonly elevated in breast cancer tissues. FSIP1 exerts an oncogenic function by cooperating with MRP1 to promote chemo-resistance. It may also function as an independent prognostic biomarker as well as a molecule to be targeted therapeutically in future breast cancer therapies.

## Supplementary information

Supplementary figures

Supplementary figure legends
