# Supplementary figures and images for: Over-expression of FSIP1 promotes breast cancer progression and confers resistance to docetaxel via MRP1 stabilization
Source: Cell Death Dis. 2019 Feb 27;10(3):204. doi: 10.1038/s41419-018-1248-8 (PMC6393503; doi:10.1038/s41419-018-1248-8)

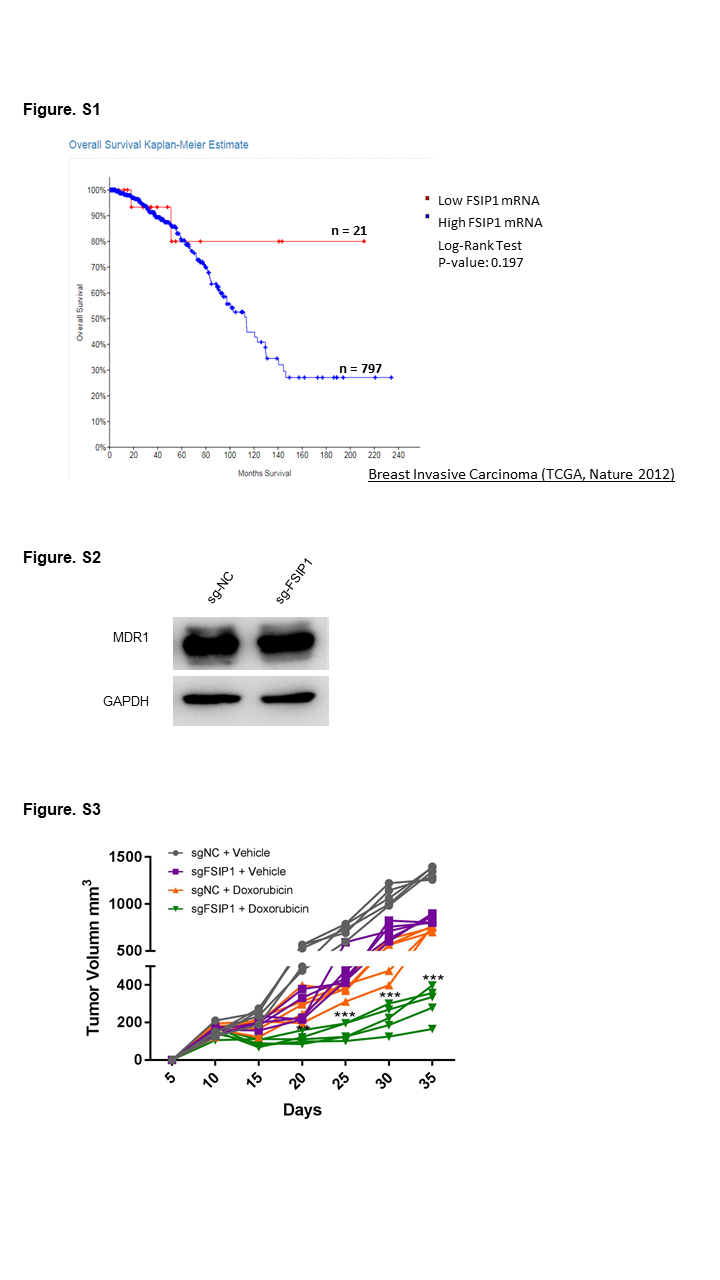

Supplement: Supplementary file 1 — Supplementary figures [file 41419_2018_1248_MOESM1_ESM.tif]
