# Supplementary material for: Over-expression of FSIP1 promotes breast cancer progression and confers resistance to docetaxel via MRP1 stabilization
Source: Cell Death Dis. 2019 Feb 27;10(3):204. doi: 10.1038/s41419-018-1248-8 (PMC6393503; doi:10.1038/s41419-018-1248-8)
Supplement: Supplementary file 2 — Supplementary figure legends [file 41419_2018_1248_MOESM2_ESM.docx]

**Figure S**1. In [Nature.](https://www.ncbi.nlm.nih.gov/pubmed/23000897) 2012 TCGA Database, there are totally 825 samples in which 2.6% patients (n=27) had FSIP1 mRNA downregulation. The patients with low FSIP1 mRNA tend to have improved overall survival.

**Figure S2.** Western blot showing knocking out FSIP1 cannot affect MDR1 expression. **Figure S3.** Tumor volumes in nude mice transplanted with control and FSIP1 knockout MDA-MB-231 cells and regression in tumor volumes post doxorubicin administration. The *** indicates a statistical significant difference in tumor volume exists between sgNC+ Doxorubicin group and sgFSIP1 + Doxorubicin group.
